# Covid-19 omicron variant infection in neonates of Guangdong province-a report of 52 cases

**DOI:** 10.3389/fped.2023.1191651

**Published:** 2023-06-20

**Authors:** Yi-Kang Yang, Fen Lin, Jian-Feng Lin, Chun-Fan Lin, Li-Li Liu, Yu-Bin Ma, Xian-Yao Wang, Yu-Wei Liao, Yu-Chan Huang, Guang-Kuan Zeng, Bei-Ru Xiao, Shan-Hua Huang, Yu-Mei Xu, Yue-E Chen, Yan-Bin Cao, Li-Ye Yang

**Affiliations:** ^1^ Institute of Medicine and Nursing, Hubei University of Medicine, Shiyan, China; ^2^ Precision Medical Lab Center, People's Hospital of Yangjiang, Yangjiang, China; ^3^ Precision Medical Lab Center, Chaozhou Central Hospital, Chaozhou, China; ^4^ Department of Neonatology, Pengpai Memorial Hospital, Shanwei, China; ^5^ Department of Neonatology, People's Hospital of Yangjiang Affiliated to Guangdong Medical University, Yangjiang, China; ^6^ Department of Neonatology, Chaozhou Central Hospital, Chaozhou, China; ^7^ Department of Pediatrics, Shantou Central Hospital, Shantou, China; ^8^ Key Laboratory of Respiratory Disease of Yangjiang, People’s Hospital of Yangjiang Affiliated to Guangdong Medical University, Yangjiang, China

**Keywords:** COVID-19, omicron, neonate, China, prognosis, SARS-CoV-2

## Abstract

**Objective:**

To analyze the clinical characteristics of neonatal infection during the outbreak of COVID-19 omicron variant in Guangdong province of China.

**Method:**

The clinical data of neonates infected with COVID-19 omicron variant were collected from three hospitals of Guangdong province, their epidemiological history, clinical manifestation and prognosis were summarized.

**Results:**

From December 12, 2022 to January 15, 2023, a total of 52 neonates with COVID-19 infection were identified across three hospitals in Guangdong Province, including 34 males and 18 females. The age of diagnosis was 18.42 ± 6.32 days. 24 cases had clear contact history with adults who were suspected to be infected with COVID-19. The most common clinical manifestation was fever (43/52, 82.7%), the duration of fever was 1–8 days. The other clinical manifestations were cough (27/52, 51.9%), rales (21/52, 40.4%), nasal congestion (10/52, 19.2%), shortness of breath (2/52, 3.8%), and vomiting (4/52, 7.7%). C-reactive protein was only increased in 3 cases. Chest radiological examination was performed in 42 neonates, twenty-three cases showed abnormal chest radiographic findings, including ground-glass opacity and consolidation. Fifty cases were admitted with COVID-19 presentation, two cases were admitted for jaundice. The hospital stay was 6.59 ± 2.77 days. The clinical classification included 3 cases of severe COVID-19 and one critical case. Fifty-one cases were cured and discharged after general treatment, and one critical case with respiratory failure was intubated and transferred to another hospital.

**Conclusion:**

The COVID-19 omicron variant infection in neonates is usually mild. The clinical manifestation and laboratory results are not specific, and the short-term prognosis is good.

## 1. Introduction

COVID-19 is an acute respiratory infectious disease, which is caused by severe acute respiratory syndrome coronavirus 2 (SARS-CoV-2), neonates are also susceptible populations for COVID-19 infection (1–4). China underwent a surge of omicron infections after abandoning “zero COVID” strategies on December 7, 2022 (5). Massive children infections including neonates are speculated. According to the report of the Chinese Center for Disease Control and Prevention (CDC), the epidemic strains are mainly omicron BA.5.2 and BF.7 (6). From December 8, 2022 to January 2023, an epidemic of omicron strain also occurred in Guangdong province. There was no report of omicron variant infection in neonates from China after this epidemic. Confirmed neonatal SARS-CoV-2 infection was uncommon over the past 3 years of the pandemic in China. Infection trend in the neonates broadly followed that seen in the general population, although at a lower level. Since the literature of the disease presentation and outcome in neonates is sparse, there is an urgent need to understand the clinical characteristics and management of neonates with community acquired COVID-19.

Here, we summarize the early clinical characteristics of COVID-19 infection in neonates identified during this outbreak in three hospitals of Guangdong province, and analyze the epidemiological history, treatment measures and prognosis of the infection, and provide reference experience for the prevention and treatment of neonatal COVID-19 omicron variant infection.

## 2. Methods

### 2.1. Study population

From December 8, 2022 to January 15, 2023, neonates admitted in Neonatal wards of People's Hospital of Yangjiang (Tertiary hospital, the biggest hospital in Yangjiang area), Chaozhou Central Hospital (Tertiary hospital with the biggest Neonate ward in Chaozhou area) and Pengpai Memorial Hospital (Secondary hospital in Shanwei area) were retrospectively reviewed. The diagnostic criteria for neonates with COVID-19 were: (1) age ≤28 days old; (2) positive for SARS-CoV-2 nucleic acid in nasal swab and (or) oropharynx swab, the presence of SARS-CoV-2 viral RNA was tested using an in-house Taqman rt-real-time PCR assay targeting N and ORF1ab genes. The date of disease onset was defined as the day a symptom was noticed. Fever was defined as a temporary increase in the body's temperature (over 37.2°C). Neonatal anemia was defined as a neonate had a lower hemoglobin (lower than 130 g/dl). Demographic and clinical records, and laboratory results were reviewed and collected by the ordering pediatricians from electronic medical records. Information recorded included date of birth, sex, weight at birth, mode of delivery, gestational age, feeding mode, Apgar score, signs, laboratory findings, medical records and underlying comorbidities. Laboratory tests including blood routine, renal and hepatic function test were also reviewed. Epidemiological history, chest imaging findings and outcomes were also collected.

This study was approved by the Ethics Committee of People's Hospital of Yangjiang (20230003), Ethics Committee of Pengpai Memorial Hospital and Chaozhou Central Hospital. As the patient's data were analyzed anonymously, a waiver of written consent was approved by the Ethics Committee of the three Hospitals.

### 2.2. Clinical diagnosis and classification

COVID-19 diagnosis and classification criteria were based on “New coronavirus pneumonia diagnosis and treatment protocol (trial version 10)”, which was issued by the national health commission of P. R. China (7). Mild infection was defined as having only mild clinical manifestations such as fever and cough but no imaging manifestations of pneumonia; the moderate type was defined as those with clinical manifestations and imaging manifestations of pneumonia; severe type was defined as persistent fever for more than 3 days, shortness of breath, hypoxemia, dyspnea, lethargy, convulsion, difficulty in feeding or refusing to eat, and obvious imaging manifestations of pneumonia; critical type was defined as respiratory failure requiring respiratory support, shock, or other systemic organ failure.

### 2.3. Discharge criteria

The clinical condition was stable and the symptoms and signs disappeared.

### 2.4. Statistical analysis

SPSS 19.0 software was used for statistical analysis. Categorical variables were reported as number and percentage, while continuous variables were shown as median and interquartile ranges (laboratory results) or as mean and standard deviation (SD).

## 3. Results

### 3.1. Demographics

**Figure 1:**
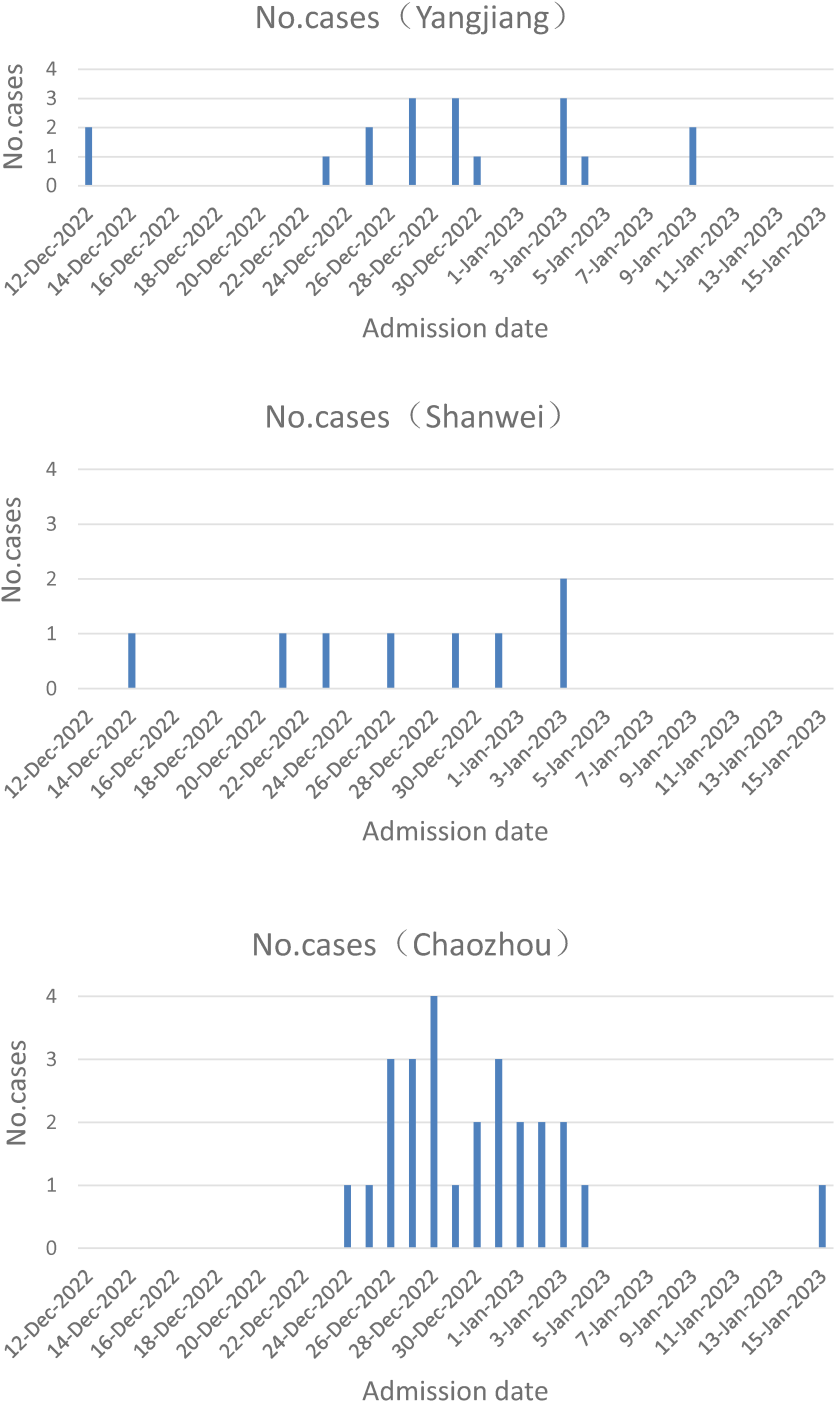
Case numbers of three hospitals at admission date.

From December 12, 2022 to January 15, 2023, fifty-two neonates with COVID-19 were admitted in the Neonatal wards of People's Hospital of Yangjiang (*n* = 18), Pengpai Memorial Hospital (*n* = 8), and Chaozhou Central Hospital (*n* = 26) of Guangdong province of China (Figure 1). They were positive for SARS-CoV-2 polymerase chain reaction (PCR) test, including 34 males and 18 females; their gestational age was 36–41 weeks and birth weight was 3.24 ± 0.316 kg; thirty cases were vaginal delivery and twenty-two cases were cesarean section; seven cases were exclusively breast feeding, thirteen cases were exclusively formula feeding, twenty-seven cases were mixed feeding and feeding pattern was unknown in 5 cases. Median age at admission was 19 days for all neonates (Table 1). Twenty-four cases had clear contact history with adults who were suspected to be infected with SARS-CoV-2 after birth.

**Table 1:** General information and clinical features of 52 newborns with omicron variant of COVID-19.

Variables	Values
Age, days at admission (*n* = 52)	
Mean ± SD	18.42 ± 6.32
Median (IQR)	19 (11–22)
≤7	2 (3.85%)
8–14	15 (28.85%)
15–21	17 (32.7%)
22–28	18 (34.62%)
Sex (*n* = 52)	
Female	18 (34.6%)
Male	34 (65.4%)
Mode of delivery (*n* = 52)	
Cesarean delivery	22 (42.3%)
Vaginal delivery	30 (57.7%)
Birthweight (kg) (*n* = 52)	
Mean ± SD	3.24 ± 0.316
Presenting features^a^ (*n* = 52)	
Fever	43 (82.7%)
Cough	27 (51.9%)
Shortness of breath or difficulty breathing	2 (3.8%)
Nasal congestion	10 (19.2%)
Vomiting	4 (7.7%)
Poor appetite	9 (17.3%)
Diarrhoea	2 (3.8%)
Seizure	1 (1.9%)
Rales	21 (40.4%)
Gestational age (weeks) (*n* = 52)	
Mean ± SD	38.67 ± 1.37
Median(IQR)	39 (38–40)
Duration of fever days (*n* = 42)	
Mean ± SD	2.52 ± 1.27
Median(IQR)	2 (2–3)
Peak temperature	
≥39°C	4 (9.5%)
<39°C	38 (90.5%)
Comorbidities^b^ (*n* = 52)	
None	24 (46.2%)
Jaundice	11 (21.2%)
Anemia	22 (42.3%)
Urinary tract infection	2 (3.8%)
Hyponatremia	2 (3.8%)
Hyperglycemia	1 (1.9%)
Gastrointestinal disorders	5 (9.6%)
Pertussis	1 (1.9%)
Encephalitis	1 (1.9%)
Convulsions	1 (1.9%)
Feeding pattern (*n* = 52)	
Breast feeding	7 (13.5%)
Formula feeding	13 (25%)
Mixed feeding	27 (51.9%)
Unknown	5 (9.6%)
Length of hospital stay, days (*n* = 52)	
Mean ± SD	6.59 ± 2.77
Median (IQR)	5 (4–6)
Range	10 h–17 d
Highest category of oxygen supplementation (*n* = 52)	
None	50 (96.2%)
Nasal prong oxygen	2 (3.8%)
Mechanical ventilation	1 (1.92%)
Antibiotic (*n*=52)	17 (32.7%)
Disease severity (*n*=52)	
Asymptomatic^c^	2 (3.8%)
Mild	24 (46.2%)
Moderate	22 (42.3%)
Severe	3 (5.77%)
Critical	1 (1.92%)
Outcomes (*n* = 52)	
Cured	51 (98.1%)
Transfer to another hospital	1 (1.9%)

Data are *n* (%), mean (SD), range, or median (IQR), unless otherwise stated.

^a^ Multiple presenting features were possible.

^b^ Multiple Comorbidities were possible.

^c^ 2 cases were admitted for jaundice, absent for syndromes of COVID-19 such as fever and cough.

### 3.2. Clinical characteristics

Upon admission, the most common clinical manifestation was fever (*n* = 43; 82.7%) among the 52 cases. During the course of the disease, the duration of fever was 1–8 days, the peak temperature was 37.4°C–40.1°C, and it was over 39°C in 4 cases. Fever was the only presentation in 14 cases. The respiratory symptoms were cough (*n* = 27, 51.9%), nasal congestion (*n* = 10, 19.2%), shortness of breath (*n *= 2, 3.8%). Lung rales could be identified in 21 cases upon auscultation. Twelve cases had digestive symptoms, including anorexia (*n* = 9, 17.3%), vomiting (*n* = 4, 7.7%), and diarrhea (*n* = 2, 3.8%). One case had fever (37.8°C) and cough for one day and was admitted in one hospital, she was treated for 4 days and discharged with normal temperature, one day later, she had fever (37.7°C) again, then she was admitted and treated for 3 days, and released without fever.

Among infants with COVID-19, thirty-two cases had at least 1 comorbidity, the most prevalent was anemia (22, 42.3%), jaundice accounted for 21.2% (11/52) of comorbidities. Gastrointestinal disorders was observed in 5 cases, and 2 cases of urinary tract infection, two cases of hyponatremia, one case of hyperglycemia, one case of pertussis and one critical case with suspected encephalitis were also identified.

One critical case of 28 days old boy was admitted for cough and respiratory distress, he had no fever, presented with persistent seizure, and his condition deteriorated rapidly, he was diagnosed as respiratory failure, encephalitis, and hyponatremia. He was intubated and received mechanical ventilation, then was transferred to another hospital for further therapy.

### 3.3. Laboratory test and chest imaging

Complete blood cell count, blood biochemistry and infection biomarkers were tested upon admission. Laboratory tests revealed leukopenia (*n* = 22, 42.3%), neutropenia (*n* = 10, 19.2%), monocytosis (*n* = 52, 100%). Lymphopenia only occurred in 3 cases. Hemoglobin was decreased in 22 cases (anemia). ALT increased in one case, and AST increased in 20 cases, they were within 2 times of upper reference limits, implied no severe liver injury in this study group. C-reactive protein (CRP) was elevated in only 3 neonates. IL-6 was slightly increased in 2 of 8 tested cases in one hospital (People's Hospital of Yangjiang) (Table 2).

**Table 2:** Laboratory results and radiological findings.

Variables	Values	Normal values
Hemoglobin, g/dl (*n* = 52), Mean ± SD	137.17 ± 21.49	130–175 g/L
median (IQR)	135 (126–146)	
<130 g/L, *n* (%)	22 (42.3%)	
Platelets, ×10⁹/L (*n* = 52), Mean ± SD	344.60 ± 97.07	125–350 × 10^9^ cells/L
Median (IQR)	347 (278–413)	
WBC count, ×10⁹/L (*n* = 52), Mean ± SD	9.00 ± 3.11	3.5–9.5 × 10^9^ cells/L
Median (IQR)	9.81 (7.93–10.89)	
>9.5 × 10⁹/L, *n* (%)	22 (42.3%)	
Neutrophil count, ×10⁹/L(*n* = 52), Mean ± SD	3.07 ± 1.66	1.8–6.3 × 10^9^ cells/L
median (IQR)	2.41 (1.9–3.79)	
Lymphocyte count, ×10⁹/L (*n* = 52), Mean ± SD	3.82 ± 2.43	1.1–3.2 × 10^9^ cells/L
median (IQR)	4 (2.41–6.35)	
<1 × 10⁹/L, *n* (%)	3 (5.8%)	
Monocyte count, ×10⁹/L (*n* = 52), Mean ± SD	1.76 ± 0.77	0.1–0.6 × 10^9^ cells/L
median (IQR)	1.53 (1.01–2.67)	
>0.6 × 10⁹/L, *n* (%)	52 (100%)	
C-reactive protein, mg/L (*n* = 52), Mean ± SD	2.70 ± 4.89	0–6 mg/L
median (IQR)	1.84 (1.2–3.21)	
>6 mg/L, *n* (%)	3 (5.8%)	
IL-6, pg/ml (*n* = 8), Range	0.49–57.19	0–5.3 pg/ml
>5.3 pg/ml	2 (25%)	
ALT, U/L (*n* = 51), Mean ± SD	21.03 ± 10.25	9–50 U/L
median (IQR)	15.4 (12–22.4)	
>50 U/L, *n* (%)	1 (1.9%)	
AST, U/L (*n* = 52), Mean ± SD	41.94 ± 22.31	15–40 U/L
median (IQR)	36.4 (30–56.4)	
>40 U/L, *n* (%)	20 (38.5%)	
LDH, U/L (*n* = 52), Mean ± SD	393.21 ± 152.61	109–450 U/L
median (IQR)	362 (339–458)	
>450 U/L, *n* (%)	4 (7.7%)	
CK, U/L (*n* = 52), Mean ± SD	152.65 ± 120.24	50–310 U/L
median (IQR)	112 (74–182)	
>310 U/L, *n* (%)	2 (3.8%)	
CK-MB, U/L (*n* = 52), Mean ± SD	25.27 ± 14.69	0–24 U/L
median (IQR)	31.8 (26.7–38.2)	
>24 U/L, *n* (%)	24 (46.2%)	
Positive chest CT finding (*n* = 21), *n* (%)	18 (85.7%)	negative
Positive chest x-ray finding (*n* = 21), *n* (%)	5 (23.8%)	negative

WBC, White blood cells; ALT, Alanine aminotransferase; AST, Aspartate transaminase; LDH, lactate dehydrogenase; CK, creatine kinase; CK-MB, Creatine Kinase Myocardial Band; CRP, C-reactive protein.

Twenty cases were also tested for another 7 common respiratory viruses (influenza virus A and B, parainfluenza virus 1, 2, and 3, respiratory syncytial virus, and adenovirus) by immunofluorescence assay, they were negative for these viruses. Blood bacteria culture was performed in 11 cases, and *hemolytic staphylococcus* was identified in one case. Respiratory specimen culture was performed in 6 cases, five cases were identified with infection of *staphylococcus aureus*.

Chest x-rays were performed in 21 infants (People's Hospital of Yangjiang and Pengpai Memorial Hospital) and pneumonia was detected in 5 of them (23.8%). Chest computed tomography (CT) was performed in 21 neonates (Chaozhou Central Hospital), abnormal chest radiographic findings, including ground-glass opacity and consolidation, were identified in 18 cases.

### 3.4. Clinical classification

COVID-19 disease was deemed by clinicians to be the primary diagnosis in 50 (96.15%) of 52 infants, while in 2 jaundiced infants (3.85%) it was an incidental diagnosis. Based on their clinical presentations, twenty-four cases were mild, twenty-two cases were moderate for their radiological chest findings, three cases met the criteria for severe COVID-19 infection and one critical case were diagnosed according to the classification of China CDC (6), and no child was diagnosed with multi-system inflammatory syndrome in children (MIS-C), as per USA case definition (8) (Table 1).

### 3.5. Treatment and prognosis

None of the 52 neonates received any antiviral drug therapy or specific therapy for SARS-CoV-2. Seventeen cases were treated with antibiotics at the beginning of the disease and terminated after bacterial infection was excluded or cured. The duration of antibiotics use was 1–5 days. Two cases presented with mild respiratory distress and need nasal oxygen inhalation, and one critical case was intubated and need mechanical ventilation. The symptoms of 51 neonates including 3 severe cases relieved after general treatment. One critical case was transferred to another hospital. The duration of hospitalization was 10 h–17 days. During hospitalization, mothers were separated from their infants, and breastfeeding was discontinued.

## 4. Discussion

Since the first half of 2022, the omicron variant was the most prevalent strain of COVID-19 in China mainland, which was characterized by its stronger infectivity and mild clinical symptoms than other strains (9, 10). China underwent a surge of omicron infections after abandoning “zero COVID” strategies on December 7, 2022 (5). Subsequently, the SARS-CoV-2 Omicron variant has quickly spread throughout China, affecting individuals of all ages. This report outlines our experience with neonates diagnosed with COVID-19 in three hospitals across Guangdong Province during the SARS-CoV-2 Omicron epidemic.

The main symptom of newborns with omicron variant infection was fever in our study cohort, and the other symptoms mainly involved respiratory and digestive system, which were similar to the clinical manifestations of other strains of neonatal infection (11). It is suggested that routine detection of SARS-CoV-2 nucleic acid may be necessary for febrile neonates during the epidemic period of omicron variant of COVID-19 (11). In addition, digestive system symptoms in this study were less, no other systems such as skin and circulatory system symptom was identified. It is suggested that the clinical manifestation of omicron strain may be different from that of other strains (12), this needs to be confirmed by a larger sample size.

A review described SARS-CoV-2 infection in neonates, totally 58 neonates were summarized with SARS-CoV-2 infection (4 cases were a congenital infection), and 29 (50%) were symptomatic (23 required ICU), respiratory symptoms was the predominant manifestation (70%) (11). No mortality was reported in SARS-CoV-2-positive neonates (11). All 52 cases in our study were symptomatic, because asymptomatic infants need not to come to see doctors. The risk of SARS-CoV-2 infection in neonates is extremely low (13, 14), one reason is that the neonates have SARS-CoV-2 antibody through vaccination of his/her mother, the other reason is the strong measures (including separation of baby and COVID-19 mother) taken to prevent perinatal period infection among high-risk groups in China (14). In our study, mothers were separated from their in-hospital infants, and breastfeeding was discontinued. There have been varying guidelines regarding the separation of mothers with COVID-19 from their infants and breastfeeding during hospitalization. Initially, some hospitals (including our hospital) recommended separating mothers with COVID-19 from their infants as a precautionary measure to prevent the transmission of the virus. However, recent evidence suggests that the risk of transmission from a mother with COVID-19 to her infant through breastfeeding is low. If appropriate measures are applied, it is not necessary to separate the neonates from his/her mother, and breast feeding is encouraged (15).

Our study neonates were 3–28 days old, nearly half of them (24/52) could trace their infection routes from their surroundings. No congenital infection was identified in our study cohort. Twenty-four neonates from SARS-CoV-2 RNA positive mothers were not infected from their mothers in the same period of one hospital (data not shown). Based on the available literature and our data, we presume that SARS-CoV-2 vertical transmission, including transplacental route, is rare, and exposed neonates generally show favorable health outcomes (16).

Lymphopenia was consistently identified in adults, and it was associated with increased disease severity of COVID-19 (17). Only 3 of 52 neonates experienced lymphopenia in our study, the absence of lymphopenia observed in neonates with COVID-19 may be due to their relatively high thymic output during this developmental stage. Additionally, the immature state of their monocytes may result in a reduced cytokine response, which is indicative of a more favorable immune system response to SARS-CoV-2. Our study further supports this by showing that neonates with COVID-19 had lower levels of IL-6. As a result, there is a reduced risk of excessive inflammation associated with severe lung injury (18, 19). Monocytosis was detected in all 52 neonates, increased monocytes may represent a physiological and immature response of the marrow of infants to a variety of exogenous stimuli including COVID-19 (20).

All 52 cases were symptomatic in this group of newborns, 3 cases were severe and one was critical. Previous reports have indicated that neonatal SARS-CoV-2 infection can result in both common and severe cases, with reported cases of mortality (15). This study is consistent with previous studies, that adults and children showed mild clinical symptoms of omicron variant infection than other counterparts (8). Most of the severe or critical cases of neonates reported in the past were complicated with premature birth, congenital malformation or other underling diseases (11, 21), while all of the severe or critical cases in our group were healthy in the past.

The treatment protocol of neonatal COVID-19 infection is mainly appropriate nursing, and anti-virus drug is not recommended (22). Fifty-one neonates in this group recovered after close monitoring and general treatment, and they presented a self-limited course of disease, and the short-term prognosis was good, but the number of breast-feeding cases was significantly lower than that before admission, which may be related to the separation of mothers and infants and the influence of mother's anxiety on lactation during the epidemic period. Reports suggest that neonatal COVID-19 could have implications for brain development, highlighting the importance of long-term follow-up and prognosis monitoring (23).

Twenty-one cases of infants received chest x-ray, and only 5 cases were identified with pneumonia; while 21 cases of infants received chest CT test, eighteen cases was complicated with pneumonia. It seemed that CT could detect more abnormal findings than that of x-ray, this is consistent to previous study from Turkey (18). However, these additional CT findings did not affect medical management. Therefore, CT is not clinically indicated for the initial evaluation of mild to moderately symptomatic infants with COVID-19 pneumonia (24). As for severe and critical cases, CT could provide detailed information for clinical decision (24).

There are several limitations to our study that must be acknowledged. First, our study only includes neonates who sought medical attention and exhibited symptoms of respiratory system and fever, and therefore does not include asymptomatic neonates or atypical cases. As a result, our case cohort may be biased towards more severe illness. Additionally, the two hospitals included in our study are tertiary hospitals, which may represent a relatively higher severity of COVID-19 cases in neonates and may not reflect the overall distribution of disease severity in neonates. Although all of the neonates were treated according to the proposal provided by the national health commission of P. R. China (7), treatment measures and follow-up among three hospitals are not identical, and designing a strict protocol would be impossible and unethical. Furthermore, most of the infants do not have a respiratory PCR panel to assess co-infection, we could not exclude the possibility of co-infection in all cases.

In summary, the Covid-19 omicron variant infection in neonates is usually mild. Some infants presented with mild symptoms but were hospitalized due to their very young age. The clinical manifestation and laboratory results are not specific, and the short-term prognosis is relatively good.

## Data Availability

The raw data supporting the conclusions of this article will be made available by the authors, without undue reservation.
